# Prognostic Impact of Gastrointestinal Immune-Related Adverse Events Depends on Nutritional Status in Cancer Patients Treated with Immune Checkpoint Inhibitors

**DOI:** 10.3390/cancers17162634

**Published:** 2025-08-12

**Authors:** Shoichiro Hirata, Yoshiyasu Kono, Emi Tanaka, Masahiko Sue, Yasuto Takeuchi, Tomoki Yoshikawa, Yoshie Maki, Tomohiro Kamio, Daisuke Kametaka, Katsunori Matsueda, Chihiro Sakaguchi, Kenta Hamada, Masaya Iwamuro, Seiji Kawano, Yoshiro Kawahara, Motoyuki Otsuka

**Affiliations:** 1 Department of Gastroenterology and Hepatology, Faculty of Medicine, Dentistry and Pharmaceutical Sciences, Okayama University, 2-5-1 Shikata-cho, Kita-ku, Okayama 700-8558, Japanpvrg6aru@s.okayama-u.ac.jp (T.Y.); pr145h2k@s.okayama-u.ac.jp (M.I.); otsukamoto@okayama-u.ac.jp (M.O.); 2 Department of Gastroenterology, Japanese Red Cross Society Himeji Hospital, 1-12-1 Shimoteno, Himeji 670-8540, Japan; 3 Department of Regenerative Medicine, Center for Innovative Clinical Medicine, Okayama University Hospital, Okayama 700-8558, Japan; 4 Department of Practical Gastrointestinal Endoscopy, Faculty of Medicine, Dentistry and Pharmaceutical Sciences, Okayama University, 2-5-1 Shikata-cho, Kita-ku, Okayama 700-8558, Japan; pidw8xq3@s.okayama-u.ac.jp (K.H.); yoshirok@md.okayama-u.ac.jp (Y.K.)

**Keywords:** gastrointestinal immune-related adverse events, immune checkpoint inhibitors, prognostic nutrition index

## Abstract

**Background:** Gastrointestinal immune-related adverse events (GI-irAEs) are recognized complications of immune checkpoint inhibitors (ICIs), but their prognostic relevance and associated risk factors remain unclear. This study aimed to assess whether baseline nutritional status, measured using the prognostic nutritional index (PNI), modifies the prognostic impact of GI-irAEs, and to identify clinical factors associated with their occurrence. **Methods:** We retrospectively analyzed 1104 cancer patients treated with ICIs at a single institution. GI-irAEs were defined as gastrointestinal symptoms requiring clinical intervention. Patients were stratified by irAE type and PNI (≥40 vs. <40), and differences in survival and treatment response were evaluated. Potential risk factors for developing GI-irAEs were also examined. **Results:** GI-irAEs occurred in 2.7% of patients and were associated with prolonged overall survival (median: 28.7 vs. 14.0 months) among those with PNI ≥ 40. This survival advantage was not observed in patients with PNI < 40. The PNI-dependent prognostic pattern was specific to GI-irAEs and not observed for non-GI irAEs. Similar trends were confirmed in 4- and 8-week landmark analyses. Differences in objective response rate and disease control rate by PNI status were most pronounced in patients with GI-irAEs. The use of anti-CTLA-4 antibodies was significantly associated with GI-irAE development (odds ratio 4.24; 95% confidence interval 1.73–10.39). **Conclusions:** GI-irAEs appear to confer a survival benefit primarily in patients with preserved nutritional status. PNI may serve as a useful tool to contextualize the clinical relevance of GI-irAEs and help identify patients most likely to benefit from immune activation during ICI therapy.

## Simple Summary

Immune checkpoint inhibitors (ICIs) can induce gastrointestinal immune-related adverse events (GI-irAEs), but their prognostic significance remains unclear. We retrospectively analyzed 1104 cancer patients treated with ICIs to investigate whether baseline nutritional status, assessed using the prognostic nutritional index (PNI), modifies the impact of GI-irAEs. GI-irAEs were associated with improved survival and treatment response, but only among patients with preserved nutritional status (PNI ≥ 40). In contrast, no such benefit was observed in patients with poor nutritional status (PNI < 40). This PNI-dependent pattern was not observed with other types of irAEs. Additionally, the use of anti-CTLA-4 antibodies was strongly associated with the development of GI-irAEs. These findings suggest that nutritional status may influence the clinical relevance of GI-irAEs and underscore the importance of nutritional assessment in patients undergoing immunotherapy.

## 1. Introduction

Recent advances in cancer therapy, particularly the advent of immune checkpoint inhibitors (ICIs), have markedly improved survival outcomes in various malignancies. However, important challenges remain, such as variable treatment responses, development of resistance, and the frequent occurrence of immune-related adverse events (irAEs), which can lead to treatment interruption or discontinuation. As emphasized by Bellan et al., a deeper understanding of host-related factors, including nutritional and immunological status, is crucial to optimize the safety and efficacy of ICI-based treatments [1]. ICIs have revolutionized the treatment landscape for various malignancies by restoring anti-tumor immune responses [2,3,4,5]. However, their use is frequently accompanied by irAEs, which can affect multiple organ systems and may lead to treatment interruption or discontinuation. Among these, gastrointestinal irAEs (GI-irAEs)—such as diarrhea and colitis—are of particular concern due to their potential severity and negative impact on patients’ nutritional status and quality of life [6,7,8,9].

While several studies have demonstrated that the occurrence of irAEs is associated with improved survival outcomes [10,11], the prognostic significance of GI-irAEs specifically remains less well established [12,13]. Furthermore, the clinical outcomes of GI-irAEs may vary depending on individual patient factors, including nutritional reserve. Malnutrition is common in patients with advanced cancer and has been shown to adversely affect both immune competence and treatment tolerance [14,15].

The prognostic nutritional index (PNI), calculated from serum albumin levels and total lymphocyte count, is a simple and validated indicator of nutritional and immunological status. Previous studies suggest that PNI may serve as a predictor of clinical outcomes in patients receiving ICIs [16,17,18]. Moreover, emerging evidence indicates that the survival benefit associated with irAEs may be influenced by the patient’s nutritional status [19,20]. However, whether nutritional status modifies the prognostic impact of GI-irAEs has not been thoroughly investigated.

Therefore, this study aimed to evaluate the influence of baseline nutritional status, assessed using the PNI, on the prognostic significance of GI-irAEs in cancer patients treated with ICIs. We also sought to identify clinical factors associated with the development of GI-irAEs. This study provides novel insights by stratifying the prognostic relevance of GI-irAEs according to host nutritional status—an often overlooked factor in previous research.

## 2. Materials and Methods

### 2.1. Study Design and Patient Selection

**Figure 1:**
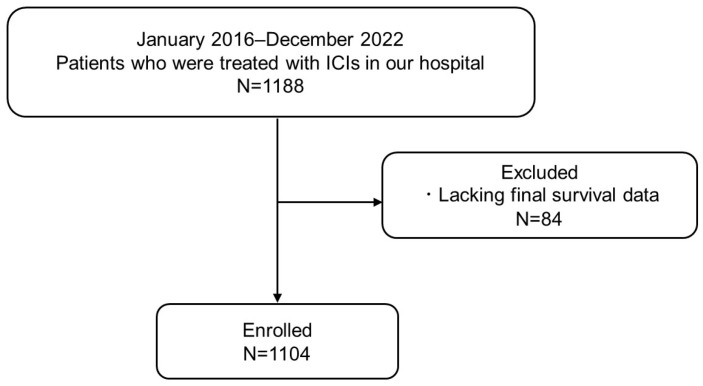
Study flow diagram showing the patient selection process for analysis. Among patients who received immune checkpoint inhibitors, those with available clinical and outcome data were included in the final cohort for evaluation of immune-related adverse events and survival. ICI, immune checkpoint inhibitor.

This retrospective cohort study included 1104 patients who initiated ICIs therapy at Okayama University Hospital between January 2016 and December 2022. Patients with sufficient clinical, laboratory, and outcome data were included for analysis of irAEs and survival outcomes. The patient selection process is summarized in the study flow diagram (Figure 1).

### 2.2. Data Collection

Demographic data (age, sex), clinical status (Eastern Cooperative Oncology Group Performance Status [ECOG PS], body mass index [BMI]), laboratory parameters (serum albumin [ALB], neutrophil-to-lymphocyte ratio [NLR], platelet-to-lymphocyte ratio [PLR]), tumor characteristics (primary site), treatment regimen (type of ICI), comorbidities, and types of irAEs were collected. The PNI was calculated using the following formula:

PNI = 10 × serum albumin (g/dL) + 0.005 × total lymphocyte count (/mm^3^).

Patients with missing baseline laboratory data required for PNI calculation, survival endpoints, or irAE status were excluded from the corresponding analyses. No data imputation was performed.

### 2.3. Definition of Gastrointestinal irAEs

GI-irAEs were defined as diarrhea or symptomatic gastritis confirmed by histological findings, in accordance with the American Society of Clinical Oncology Clinical Practice Guidelines and were graded using the Common Terminology Criteria for Adverse Events (CTCAE) version 5.0 [21]. All cases were reviewed and confirmed by both gastroenterologists and oncologists. When GI-irAEs were suspected, patients were evaluated by the institutional irAE team. To exclude alternative causes of gastrointestinal symptoms, stool tests for Clostridioides difficile toxin and cytomegalovirus antigenemia were performed when necessary. Abdominal computed tomography was conducted in all cases, and endoscopic examinations with histopathological evaluation were actively pursued when feasible. GI-irAE diagnoses were made based on a comprehensive assessment of clinical, radiological, endoscopic, and pathological findings.

### 2.4. Outcomes and Statistical Analysis

The primary endpoint was to evaluate the impact of GI-irAEs on overall survival (OS), overall response rate (ORR), and disease control rate (DCR), stratified by PNI status. Secondary endpoints included the association between clinical variables and OS according to PNI status, as well as the identification of risk factors for the onset of GI-irAEs. ORR and DCR were assessed according to RECIST version 1.1.

To identify factors associated with the development of GI-irAEs, we performed univariate and multivariate logistic regression analyses. OS was assessed using Cox proportional hazards models. For continuous variables such as PNI, NLR, and PLR, optimal cutoff values were determined using receiver operating characteristic curve analysis, with thresholds selected to maximize the Youden index. Because the distributions of continuous variables were non-normal, they were summarized as medians with interquartile ranges. Kaplan–Meier survival curves were used to compare OS based on irAE type and nutritional status. To minimize immortal time bias, landmark analyses were conducted at 4 and 8 weeks after initiation of ICI therapy, including only patients who survived beyond each respective time point. All statistical tests were two-tailed, and a *p*-value of <0.05 was considered statistically significant. Statistical analyses were performed using JMP Pro 17 software (SAS Institute Inc., Cary, NC, USA).

## 3. Results

### 3.1. Patient Characteristics

Between January 2016 and December 2022, a total of 1188 patients received ICIs at our hospital. After excluding 84 patients due to missing survival data, 1104 patients were included in the final analysis (Figure 1).

The baseline characteristics of these 1104 patients are summarized in Table 1. The median age was 69 years (interquartile range: 60–75), and 68% were male. Most patients had favorable performance status (ECOG PS 0–1:94%). The most common primary tumor sites were the lung (32%) and gastrointestinal (GI) tract (19%), followed by urinary tract (13%) and head and neck (13%). Anti-PD-1 antibodies were the most frequently used ICIs (72%), and 6.8% of patients received combination (doublet) ICI therapy. Common comorbidities included hypertension (35%), diabetes mellitus (18%), and chronic obstructive pulmonary disease (5.3%). Overall, 2.7% of patients experienced GI-irAEs, while 25% developed non-GI irAEs.

### 3.2. Impact of Gastrointestinal-Immune-Related Adverse Events on Prognosis by Prognostic Nutrition Index Status

**Figure 2:**
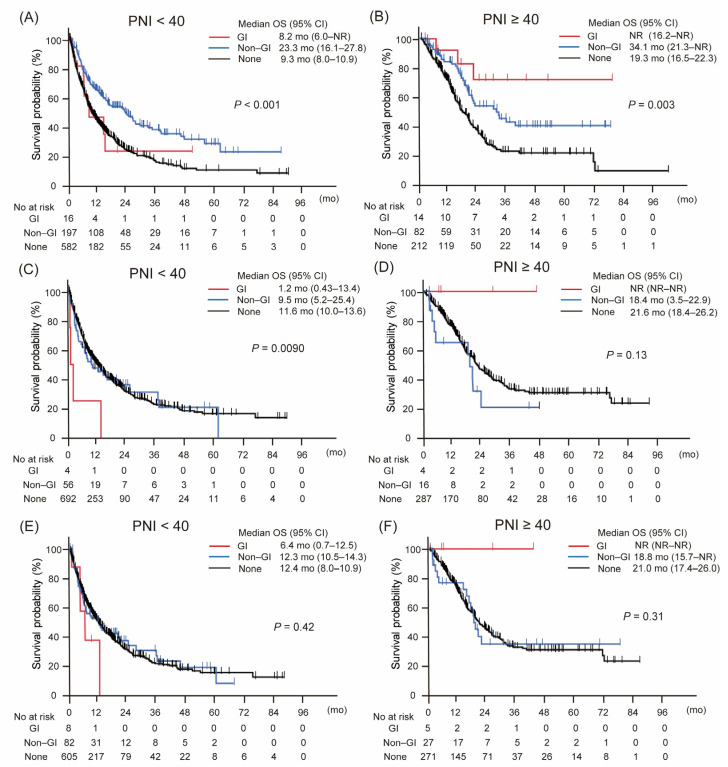
Kaplan–Meier curves of overall survival according to the type of immune-related adverse event, stratified by prognostic nutritional index (PNI). (**A**,**B**) Overall survival without landmark analysis. (**C**,**D**) Overall survival with 4-week landmark analysis. (**E**,**F**) Overall survival with 8-week landmark analysis. Gastrointestinal immune-related adverse events (GI-irAEs) demonstrated distinct impacts on overall survival depending on PNI status, unlike other types of irAEs. This pattern was consistently observed across all three analytical approaches, indicating a robust and reproducible association. Abbreviations: CI, confidence interval; GI, gastrointestinal; mo, months; No, number; NR, not reached; OS, overall survival; PNI, prognostic nutritional index.

Figure 2 presents Kaplan–Meier survival curves stratified by irAE type and PNI status, including analyses without landmark adjustment as well as 4-week and 8-week landmark analyses. Among patients without landmark analysis, those with PNI ≥ 40 who developed GI-irAEs showed the longest overall survival (OS), followed by those with non-GI irAEs and those without irAEs (Figure 2B). In contrast, among patients with PNI < 40, this survival pattern was less evident, with only non-GI irAEs associated with a modest survival benefit (Figure 2A).

This trend remained largely consistent across the landmark analyses. In the 4-week landmark analysis, although the survival advantage of GI-irAEs in the PNI ≥ 40 group did not reach statistical significance, a similar ordering was observed, with patients experiencing GI-irAEs showing the most favorable outcomes, followed by those with non-GI irAEs and those without irAEs (Figure 2D). Among patients with PNI < 40, survival differences by irAE type remained minimal (Figure 2C).

Similarly, the 8-week landmark analysis supported the findings of the non-landmark analysis: GI-irAEs continued to be associated with the most favorable survival in the PNI ≥ 40 group, while non-GI irAEs conferred modest benefits, and no clear differences were seen in the PNI < 40 group (Figure 2E,F).

These results suggest that the prognostic significance of GI-irAEs depends on both baseline nutritional status and the timing of the survival assessment.

### 3.3. Impact of Gastrointestinal-Immune-Related Adverse Events on Treatment Response by Prognostic Nutrition Index Status

**Figure 3:**
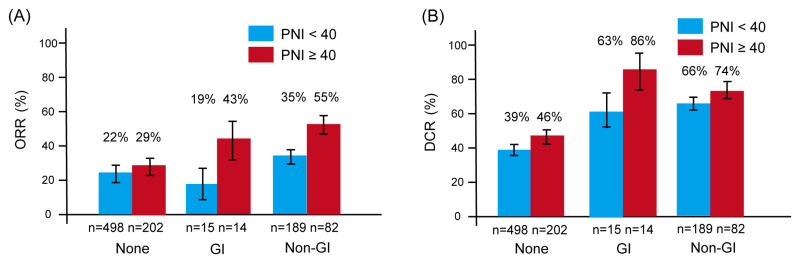
Overall response rate (ORR) and disease control rate (DCR) according to the type of immune-related adverse events (irAEs), stratified by prognostic nutritional index (PNI). Bar graphs show ORR (**A**) and DCR (**B**). Red bars indicate patients with PNI ≥ 40; blue bars indicate those with PNI < 40. Error bars represent the standard error. Both ORR and DCR were generally higher in patients who experienced irAEs, particularly among those with higher PNI. Notably, among patients with gastrointestinal irAEs (GI-irAEs), those with PNI ≥ 40 demonstrated markedly better ORR and DCR compared to those with PNI < 40, underscoring the potential modifying effect of nutritional status on treatment response. Abbreviations: DCR, disease control rate; GI, gastrointestinal; ORR, overall response rate; PNI, prognostic nutritional index.

Figure 3 shows the ORR and DCR stratified by irAE type and PNI status. For ORR (Figure 3A), the difference between patients with PNI ≥ 40 and those with PNI < 40 was most pronounced among those who developed GI-irAEs—43% versus 19%, respectively. In contrast, the PNI-related difference was smaller among patients with non-GI irAEs (55% vs. 35%) and minimal among those without irAEs (29% vs. 22%).

A similar trend was observed for DCR (Figure 3B). Among patients with GI-irAEs, those with PNI ≥ 40 had a substantially higher DCR than those with PNI < 40 (86% vs. 63%). In comparison, the difference in DCR by PNI status was less pronounced for non-GI irAEs (74% vs. 66%) and negligible among patients without irAEs (46% vs. 39%).

These findings suggest that the impact of PNI on treatment response is most evident in patients who develop GI-irAEs, whereas its influence appears limited in other subgroups.

### 3.4. Prognostic Factors for Overall Survival According to Nutritional Status

Associations between clinical variables and OS are summarized in Table 2. Good performance status (ECOG PS 0–1) was consistently associated with longer OS across all PNI strata and analysis types. Among patients with PNI < 40, an elevated neutrophil-to-lymphocyte ratio (NLR ≥ 3.1) was significantly associated with shorter OS in both the non-landmark and landmark analyses.

Non-GI irAEs were strongly associated with improved OS regardless of PNI group or landmark adjustment, with the most pronounced effect observed in the PNI < 40 subgroup (hazard ratio [HR] 0.54, 95% confidence interval [CI] 0.44–0.68; *p* < 0.0001 in the non-landmark model). In contrast, GI-irAEs demonstrated a distinct pattern: a significant survival benefit was observed only in patients with PNI ≥ 40 in the non-landmark analysis (HR 0.26, 95% CI 0.083–0.83; *p* = 0.023), while no clear benefit was seen in those with PNI < 40.

Although statistical significance was not maintained in the landmark-adjusted models—possibly due to limited sample size—the divergent trends in OS associated with GI-irAEs between high and low PNI groups remained evident. These findings suggest that while non-GI irAEs are consistent prognostic indicators regardless of nutritional status, the impact of GI-irAEs on OS appears to be modified by baseline nutrition, showing a differential effect depending on PNI level.

### 3.5. Risk Factors for Gastrointestinal Immune-Related Adverse Events

Risk factors for GI-irAEs are summarized in Table 3. In univariate analyses, PNI ≥ 40 was significantly associated with an increased risk of GI-irAEs (OR 2.3, 95% CI 1.1–4.8; *p* = 0.025), whereas PLR ≥ 1.8 was associated with a decreased risk (OR 0.45, 95% CI 0.21–0.97; *p* = 0.042). The use of anti-CTLA-4 antibodies (OR 4.3, 95% CI 1.9–10.0; *p* = 0.0006) and ICI doublet therapy (OR 3.6, 95% CI 1.4–9.2; *p* = 0.0063) were also significantly associated with the occurrence of GI-irAEs. In multivariate analysis, anti-CTLA-4 antibody use remained an independent risk factor (OR 9.2, 95% CI 1.9–45.0; *p* = 0.0062), while the associations for PNI and PLR were attenuated and did not reach statistical significance (*p* = 0.052 and *p* = 0.053, respectively).

These findings identify anti-CTLA-4 therapy as a strong predictor of GI-irAEs and suggest that host-related factors, such as nutritional and inflammatory status, may also contribute to the development of these events.

## 4. Discussion

This study investigated the clinical significance of GI-irAEs in 1104 patients treated with ICIs, with a particular focus on the role of nutritional status. GI-irAEs were associated with improved survival and treatment response, especially among patients with higher PNI scores. In addition, the use of anti-CTLA-4 antibodies was significantly associated with the development of GI-irAEs. These findings suggest that nutritional status should be routinely assessed, particularly in patients receiving anti-CTLA-4-based regimens, where GI toxicity may have a greater clinical impact.

Although irAEs are often regarded as adverse events that necessitate immunosuppressive treatment or therapy interruption, growing evidence suggests that their occurrence may reflect enhanced immune activation and correlate with favorable clinical outcomes [22,23,24]. However, most existing studies have evaluated irAEs as a whole, and relatively few have specifically examined GI-irAEs. In this context, our study provides novel and clinically relevant evidence that GI-irAEs are associated with improved survival outcomes—particularly in patients with adequate nutritional reserves.

A key aspect of our analysis was the incorporation of host nutritional status, as assessed by PNI. Malnutrition is common in patients with advanced cancer and can impair both immune function and treatment tolerance [14,15]. In our study, the survival benefit associated with GI-irAEs was observed primarily in patients with PNI ≥ 40, whereas no such benefit was seen in those with lower PNI. This finding may be attributed to the immunological consequences of malnutrition. Nutritional deficiency has been linked to impaired cell-mediated immunity, reduced lymphocyte counts, and dysregulated cytokine production—factors that can weaken the anti-tumor immune response elicited by ICIs [25,26,27]. In contrast, patients with adequate nutritional reserves may be capable of mounting a more robust immune response, and the occurrence of GI-irAEs in these individuals may reflect heightened systemic immune activation that enhances treatment efficacy. These observations suggest that sufficient nutritional status may be a prerequisite for realizing the full therapeutic benefit of GI-irAEs.

In view of these findings, we suggest that patients initiating ICI therapy with borderline or compromised nutritional status may benefit from early consultation with nutrition specialists. Although specific interventions were not evaluated in this study, incorporating basic nutritional support measures, such as ensuring adequate protein-energy intake and addressing inflammation-related malnutrition, may help optimize immunotherapy tolerance and outcomes. These considerations could be especially relevant for patients at higher risk of developing GI-irAEs, and merit further evaluation in prospective interventional studies.

Previous studies have generally reported that the development of irAEs is associated with better survival outcomes in patients receiving ICIs [10,11]. Some reports have also suggested a prognostic benefit of GI-irAEs [12,13], but the findings have been inconsistent, and most prior analyses did not stratify patients by nutritional status. Our results extend this body of evidence by demonstrating that the prognostic value of GI-irAEs is significantly influenced by baseline nutritional reserve. Among patients with poor nutritional status (PNI < 40), the occurrence of GI-irAEs did not confer a survival benefit. This discrepancy may help explain the inconsistent results of previous studies and underscores the importance of incorporating host-related factors, such as nutritional status, into irAE-outcome research.

Interestingly, this PNI-dependent effect was not observed with non-GI irAEs. One possible explanation is that GI-irAEs directly impair gastrointestinal function, resulting in nutrient loss, malabsorption, or systemic inflammation—factors that disproportionately affect patients with already compromised nutritional status. In contrast, non-GI irAEs, such as endocrine or dermatologic events, may have less impact on nutritional pathways, leading to a more consistent prognostic effect regardless of PNI. These findings suggest that the clinical significance of irAEs cannot be generalized across organ systems and should be interpreted in the context of host-related factors.

We also identified the use of anti-CTLA-4 antibodies as a significant risk factor for the development of GI-irAEs. This is consistent with previous studies reporting higher rates of colitis and diarrhea with CTLA-4 blockade, likely due to enhanced T-cell activation and reduced regulatory T-cell (Treg) function in the gut mucosa [28,29,30]. Although PNI and PLR were not statistically significant in our analysis, they may still reflect a proinflammatory or immunologically fragile state that predisposes patients to mucosal immune injury. However, in the multivariate logistic regression model, predictors such as PNI and PLR showed wide confidence intervals, suggesting potential instability in the estimates. This may be due to the relatively small number of GI-irAE events, and the number of variables included in the model. These results should therefore be interpreted with caution due to the risk of model overfitting. Further validation in larger, independent cohorts is needed to clarify the contribution of host-related factors to the development of GI-specific toxicities.

This study has several limitations. First, its retrospective design and registry-based data collection may introduce selection and reporting biases. Prospective validation is warranted to confirm the prognostic relevance of GI-irAEs in the context of nutritional status. Future studies incorporating more comprehensive and dynamic measures of nutritional status—such as body composition analysis, dietary intake assessment, or inflammation-related biomarkers—may offer deeper insights into the interplay between nutrition, immunity, and ICI efficacy. These efforts could help refine risk stratification and guide supportive care strategies for patients undergoing immunotherapy. Second, irAEs were identified based on clinical documentation without centralized adjudication. Third, although PNI is a practical and validated index, it does not capture all dimensions of nutritional status, such as muscle mass or micronutrient deficiencies. Fourth, the distribution of cancer types was unbalanced, with relatively few patients with urinary tract or head and neck cancers. This may limit the generalizability of our findings to underrepresented tumor types. Further investigations focusing on specific cancer types or including larger and more diverse populations are warranted to validate our observations. Nevertheless, our findings provide novel and clinically relevant insights into the prognostic implications of GI-irAEs and the modifying role of nutritional status in patients receiving ICIs.

## 5. Conclusions

GI-irAEs were observed in 2.7% of patients and were associated with significantly prolonged overall survival, particularly among those with preserved nutritional status (PNI ≥ 40; median OS: 28.7 vs. 14.0 months). This survival benefit was not seen in patients with low PNI. Notably, this PNI-dependent prognostic effect was observed only for GI-irAEs and not for non-GI irAEs, suggesting a unique interaction between nutritional reserve and GI-specific immune toxicity. These findings highlight the importance of evaluating host-related factors, such as nutritional status, in the management of ICI therapy. Prospective studies are warranted to confirm these observations and to determine whether nutritional optimization can improve immunotherapy outcomes.

## Figures and Tables

**Table 1:** Baseline demographic and clinical characteristics of patients who received immune checkpoint inhibitors. This table summarizes patient age, sex, performance status, body composition, laboratory parameters, cancer types, comorbidities, and treatment regimens for the entire cohort. These variables were analyzed in relation to the development of gastrointestinal immune-related adverse events (GI-irAEs) and survival outcomes.

	N = 1104
Age, years, median (IQR)	69 (60–75)
Sex, n (Male/Female)	754/350
ECOG PS, n (0/1/2/3)	526/509/63/6
BMI, kg/m^2^, median (IQR)	22 (19–24)
ALB, g/dL, median (IQR)	3.7 (3.2–4)
NLR, median (IQR)	3.0 (1.8–4.7)
PLR, median (IQR)	1.9 (1.3–3.0)
Primary tumor site, n (%)	
Lung	348 (32)
GI	209 (19)
Urinary	145 (13)
Head and Neck	141 (13)
Skin	123 (11)
Liver	56 (5.1)
Gynecology	30 (2.7)
Breast	21 (1.9)
Other	31 (2.8)
Types of ICI, n (%)	
Anti-PD-1 antibody	800 (72)
Anti-PD-L1 antibody	213 (19)
Anti-CTLA-4 antibody	91 (8.2)
Doublet	75 (6.8)
Comorbidities, n (%)	
DM	198 (18)
HT	386 (35)
Cardiovascular disease	85 (7.7)
Cerebrovascular disease	49 (4.4)
COPD	58 (5.3
CKD	29 (2.6)
Types of irAE, n (%)	
None	794 (72)
GI	30 (2.7)
Non-GI	280 (25)

Abbreviations: ECOG PS, Eastern Cooperative Oncology Group Performance Status; BMI, body mass index; ALB, albumin; NLR, neutrophil-to-lymphocyte ratio; PLR, platelet-to-lymphocyte ratio; GI, gastrointestinal; ICI, immune checkpoint inhibitor; PD-1, programmed cell death protein 1; PD-L1, programmed death-ligand 1; CTLA-4, cytotoxic T-lymphocyte-associated antigen 4; DM, diabetes mellitus; HT, hypertension; COPD, chronic obstructive pulmonary disease; CKD, chronic kidney disease.

**Table 2:** Multivariable Cox regression analysis of clinical factors associated with overall survival in patients treated with immune checkpoint inhibitors. Hazard ratios (HRs) and 95% confidence intervals (CIs) were calculated for the overall cohort and subgroups stratified by prognostic nutritional index (PNI ≥ 40 or <40), with additional landmark analyses at 4 and 8 weeks. Key prognostic factors evaluated included irAE occurrence, nutritional status, and inflammatory markers.

	PNI ≥ 40		PNI < 40	
	HR (95% CI)	*p* Value	HR (95% CI)	*p* Value
Without landmark				
ECOG PS 0–1	0.35 (0.16–0.75)	0.0070	0.35 (0.27–0.47)	<0.0001
PLR ≥ 1.8	0.97 (0.65–1.4)	0.87	0.92 (0.73–1.2)	0.50
NLR ≥ 3.1	1.2 (0.81–1.8)	0.35	1.4 (1.1–1.8)	0.0018
GI-irAE	0.26 (0.083–0.83)	0.023	0.98 (0.52–1.8)	0.95
Non-GI-irAE	0.56 (0.38–0.82)	0.0027	0.54 (0.44–0.68)	<0.0001
4w landmark				
ECOG PS 0–1	0.28 (0.13–0.61)	0.0013	0.37 (0.27–0.51)	<0.0001
PLR ≥ 1.8	0.92 (0.62–1.4)	0.68	0.93 (0.74–1.8)	0.56
NLR ≥ 3.1	1.3 (0.85–1.9)	0.25	1.4 (1.1–1.7)	0.0073
GI-irAE	1.9 × 10^−9^ (0–NR)	1.0	4.0 (1.5–11)	0.0063
Non-GI-irAE	1.6 (0.83–3.0)	0.16	1.0 (0.72–1.4)	0.91
8w landmark				
ECOG PS 0–1	0.28 (0.13–0.59)	0.0010	0.46 (0.32–0.66)	<0.0001
PLR ≥ 1.8	0.94 (0.63–1.4)	0.78	0.99 (0.78–1.3)	0.96
NLR ≥ 3.1	1.3 (0.84–1.9)	0.26	1.3 (1.0–1.7)	0.037
GI-irAE	1.9 × 10^−9^ (0–NR)	1.0	1.8 (0.74–4.4)	0.20
Non-GI-irAE	1.1 (0.62–1.8)	0.83	0.93 (0.69–1.3)	0.65

Abbreviations: CI, confidence interval; COPD, chronic obstructive pulmonary disease; ECOG PS, Eastern Cooperative Oncology Group Performance Status; GI-irAE, gastrointestinal immune-related adverse event; HR, hazard ratio; ICI, immune checkpoint inhibitor; NLR, neutrophil-to-lymphocyte ratio; PLR, platelet-to-lymphocyte ratio; PNI, prognostic nutritional index.

**Table 3:** Risk factors for gastrointestinal immune-related adverse events by logistic regression analysis OR, odds ratio; CI, confidence interval; PNI, prognostic nutritional index; GI-irAE, gastrointestinal immune-related adverse event.

	Univariate		Multivariate	
	OR (95% CI)	*p* Value	OR (95% CI)	*p* Value
Age ≥ 65	1.0 (0.47–2.1)	1.0		
Sex	1.7 (0.80–3.5)	0.17		
ECOG PS 0–1	8.8 × 10^5^ (0–NR)	0.98		
BMI ≥ 30	3.1 (0.90–11)	0.072		
PNI ≥ 40	2.3 (1.1–4.8)	0.025	2.1 (0.99–4.5)	0.052
NLR ≥ 3.1	0.57 (0.26–1.2)	0.15		
PLR ≥ 1.8	0.45 (0.21–0.97)	0.042	0.46 (0.21–1.0)	0.053
Primary site (GI)	0.65 (0.22–1.9)	0.43		
Anti-CTLA-4 antibody	4.3 (1.9–10)	0.0006	9.2 (1.9–45)	0.0062
ICI doublet	3.6 (1.4–9.2)	0.0063	0.42 (0.073–2.4)	0.33
DM	0.50 (0.15–1.7)	0.26		
HT	1.7 (0.80–3.4)	0.18		
Cardiovascular disease	0.41 (0.055–3.0)	0.38		
Cerebrovascular disease	0.74 (0.098–5.5)	0.77		
COPD	2.9 (0.98–8.6)	0.055		
CKD	1.18 × 10^−6^ (0–NR)	0.99		

## Data Availability

Due to the nature of this research, participants of this study did not agree for their data to be shared publicly, so supporting data is not available.

## Abbreviations

The following abbreviations are used in this manuscript:

ALB	Albumin
BMI	Body Mass Index
CI	Confidence Interval
CTCAE	Common Terminology Criteria for Adverse Events
DCR	Disease Control Rate
ECOG PS	Eastern Cooperative Oncology Group Performance Status
GI	Gastrointestinal
GI-irAE(s)	Gastrointestinal Immune-Related Adverse Event(s)
HR	Hazard Ratio
ICI(s)	Immune Checkpoint Inhibitor(s)
irAE(s)	Immune-Related Adverse Event(s)
mo	Months
NLR	Neutrophil-to-Lymphocyte Ratio
NR	Not Reached
OR	Odds Ratio
ORR	Overall Response Rate
OS	Overall Survival
PNI	Prognostic Nutritional Index
PLR	Platelet-to-Lymphocyte Ratio
RECIST	Response Evaluation Criteria in Solid Tumors
